# Magnetic resonance imaging of posterolateral plica of the elbow joint: Asymptomatic vs. symptomatic subjects

**DOI:** 10.1371/journal.pone.0174320

**Published:** 2017-06-16

**Authors:** Sang-Hee Choi, Suk Kyeong Ji, Seung Ah Lee, Min Jong Park, Moon Jong Chang

**Affiliations:** 1 Department of Radiology, Samsung Medical Center, Sungkyunkwan University School of Medicine, Seoul, Republic of Korea; 2 Department of Physical Medicine and Rehabilitation, College of Medicine, Kyung Hee University, Seoul, Republic of Korea; 3 Department of Orthopedic Surgery, Samsung Medical Center, Sungkyunkwan University School of Medicine, Seoul, Republic of Korea; 4 Department of Orthopedic Surgery, Seoul National University College of Medicine, Seoul National University Boramae Hospital, Seoul, Republic of Korea; Harvard Medical School/BIDMC, UNITED STATES

## Abstract

**Background:**

Magnetic resonance imaging (MRI) may be useful to diagnose a posterolateral plica syndrome of the elbow joint because this syndrome has less clear clinical features. The purposes of this study were to document mediolateral and sagittal dimensions of a posterolateral synovial fold and to determine the proportion of subjects with the posterolateral plica in asymptomatic elbows. We also aimed to determine whether the dimensions of the posterolateral synovial fold and the prevalence of the plica differ between symptomatic and asymptomatic subjects.

**Materials and methods:**

This retrospective review of prospectively collected data included 50 asymptomatic elbows (asymptomatic group) and 14 elbows with arthroscopically confirmed posterolateral plicae (plica group). The mediolateral and sagittal dimensions of the posterolateral synovial fold were measured. In addition, the criteria for the prevalence of posterolateral plica was determined with conventional MRI as synovial fold dimension ≥ 3 mm and coverage of radial head by synovial fold ≥ 30%.

**Results:**

The plica group showed larger posterolateral synovial fold dimensions compared to the asymptomatic group. The median mediolateral and sagittal dimensions of the synovial fold in the asymptomatic group were 3.8 mm and 4.7 mm, respectively. Dimensions in the plica group were 7.0 mm and 7.4 mm, respectively. When the presence of posterolateral plica was determined using the dimension criteria, there was no difference in the prevalence of the plica between the asymptomatic and the plica group. However, using the coverage criteria, the prevalence of posterolateral elbow plica was significantly greater in the plica group than the asymptomatic group (64% vs. 18%; p < 0.001).

**Conclusions:**

The patients who underwent arthroscopic surgery for posterolateral plica syndrome had larger dimensions of the posterolateral synovial fold and higher prevalence of the posterolateral plica on conventional MRI compared to the asymptomatic subjects.

## Introduction

Magnetic resonance imaging (MRI) may be useful in patients with elbow pain with less clear clinical features [1, 2]. Among the diseases featuring lateral elbow symptoms, clinical features of the lateral epicondylitis are typical. Thus, the diagnosis is relatively straightforward [3]. In contrast, in patients with posterolateral plica syndrome, symptoms and/or findings on physical examination are inconsistent [1, 4, 5]. In a previous study, the proportion of the patients with typical painful snapping on the flexion pronation test was reported as only 25% (3 of 12 patients) [1]. Thus, making a diagnosis of the posterolateral plica syndrome solely by a patient’s clinical manifestation is difficult. In this condition, radiographic assessment using MRI may be useful to make a diagnosis and to determine treatment plans. However, the presence of a prominent posterolateral synovial fold on MRI can be observed in asymptomatic subjects [2, 6, 7]. Therefore, the detailed assessment of dimensions of posterolateral synovial fold in asymptomatic subjects and comparison of the data to those of patients with pathologic posterolateral plica is useful to establish diagnostic criteria on MRI.

We sought to document the dimensions of posterolateral synovial fold and the proportion of elbows with posterolateral plica on conventional MRI using 50 asymptomatic subjects. In addition, we aimed to determine whether the dimensions of the posterolateral synovial fold and the prevalence of the plica on MRI differ between asymptomatic subjects and the patients with arthroscopically confirmed posterolateral plicae. We hypothesized that the prevalence of posterolateral plica of the elbow is low in the asymptomatic subjects. We also hypothesized that the elbows with symptomatic posterolateral plicae have larger dimensions of synovial fold and more frequently have plica on MRI compared to the asymptomatic subjects.

## Materials and methods

The study protocol was approved by the institutional review board (IRB) of the authors’ hospital. All patients gave their written informed consent for using and assessing their data. IRB of Samsung Medical Center number: 2011-03-089". This retrospective review of prospectively collected data included 50 asymptomatic elbows and 14 elbows with arthroscopically confirmed posterolateral plica syndrome. For the group of asymptomatic subjects (asymptomatic group), 25 volunteers were recruited from June 2009 to April 2011. All volunteers were male with a mean age of 22 years (range, 20–24 years). All subjects had no history of trauma, pain, or deformity in their elbow joints. In addition, they did not show any positive finding on physical examination (flexion-pronation test) [8]. There was no abnormal incidental finding on MRI of the asymptomatic group. On the other hand, arthroscopic surgery for posterolateral plica syndrome of the elbow joint was performed at our institution for 19 patients (20 elbows) from November 2005 to July 2011. Of these, six elbows were excluded for concomitant osteoarthritis (n = 3) and no available preoperative MRI (n = 3). After these exclusions, 14 patients (14 elbows) were included in the plica group. The 14 patients included 12 male and 2 female patients with a mean age of 32 years (range, 16 years to 55 years). During the arthroscopic surgery, two elbows showed chondromalacia of radial head and another two elbows had concomitant loose bodies in the joint.

The MRI examinations were performed using a 3-T MR scanner (Ingenia, Philips Healthcare, Best, The Netherlands) using a surface coil. Our routine imaging protocol included axial T1-weighted and T2-weighted images; coronal T2-weighted SPAIR and proton density weighted fat suppression images; sagittal proton density weighted fat suppression image. T1-weighted images were obtained using a spin-echo sequence with 450–810/10–20 (TR /TE range), 256–512×256–512 matrix, 12–14 cm field of view, 1–2 excitations, and 3–4-mm slice thickness with 10% spacing. T2-weighted images were obtained using SPAIR sequence with a 5300–5516/66–80 (TR/TE range; inversion time, 150 msec), 12–14 cm field of view, 1 to 2 excitations, and a 3–4-mm slice thickness with 10% spacing. Proton density weighted fat suppression image was obtained with 3800-4000/20-30 (TR/TE range), 12–14 cm field of view, 1–2 excitations, and 3–4-mm slice thickness. Imaging was performed from 10 cm above the elbow to the bicipital tuberosity. No intravenous or intra-articular contrast was administered.

**Fig 1:**
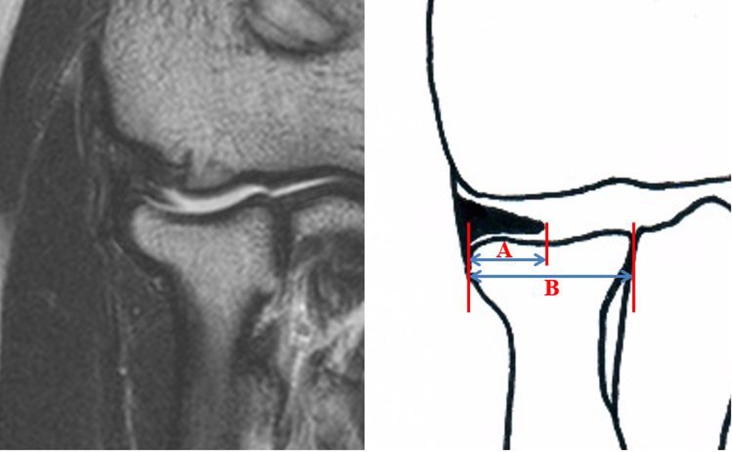
The mediolateral dimension of the synovial fold in the coronal plane was defined as the distance between lateral cortical margin of the radial head and free edge of the synovial fold (A). In addition, the mediolateral dimension of the radial head was measured using the greatest distance between medial and lateral cortical margins on the coronal images (B).

**Fig 2:**
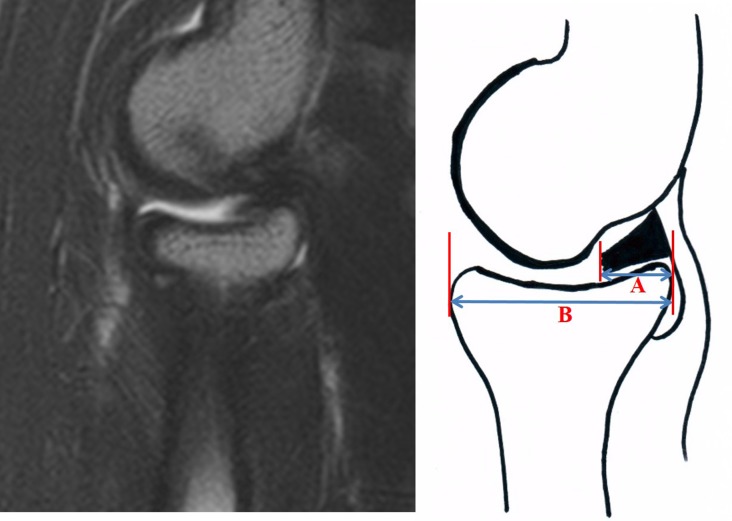
The dimension of the synovial fold in the sagittal plane was defined as the distance between posterior cortical margin of the radial head and free edge of the synovial fold (A). In addition, the sagittal dimension of the radial head was measured using the greatest distance between anterior and posterior cortical margin on the sagittal images (B).

MR evaluation was performed by two independent investigators. One interpreter was a professor and a board-certified radiologist who had undergone fellowship training. The other interpreter was an orthopedic surgeon who is a professor who had undergone fellowship training of sports medicine. The mediolateral dimension of the synovial fold in the coronal plane was defined as the distance between lateral cortical margin of the radial head and free edge of the synovial fold (Fig 1) [2]. A coronal section of the images around the pseudodefect of the capitellum with the largest dimension of synovial fold was selected to measure the dimension of posterolateral aspect of the synovial fold. On the other hand, the dimension of the synovial fold in the sagittal plane was defined as the distance between posterior cortical margin of the radial head and free edge of the synovial fold (Fig 2) [2]. A sagittal image where the plica was the largest in dimension was selected to measure the sagittal dimension of the synovial fold. In addition, the mediolateral dimension of the radial head was measured using the greatest distance between medial and lateral cortical margins on the coronal images. The sagittal dimension of the radial head was measured using the greatest distance between anterior and posterior cortical margins on the sagittal images (Figs 1 and 2). These were used to calculate the coverage of the radial head by the synovial fold in the coronal and sagittal images. All radiographic measurements were performed using a picture archiving and communication system (PACS; General Electric Medical Systems, Milwaukee, WI). Minimum detectable distance was 0.1 mm for length measurements.

Statistical analyses were performed using SPSS for Windows (version 18.0; SPSS Inc., Chicago, IL), and p-values < 0.05 were considered statistically significant. The dimension of the synovial fold was described using median and interquartile range. Then, the dimensions of the synovial folds were compared between the asymptomatic group and the plica group. The statistical significance was determined using Mann-Whitney test. We set the presence of posterolateral plica at a mediolateral or sagittal dimension >3 mm (dimension criteria). We set this criteria by applying the criterion which was used to assess craniocaudal dimension in the previous study [2]. In addition, when the coverage of the radial head by the synovial fold was ≥30%, we defined the synovial fold as the plica according to the previous study (coverage criteria) [9]. The prevalence of the posterolateral plica was compared between the asymptomatic group and the plica group. The statistical significance was determined using the Chi-square test. To determine the intra- and interobserver reliabilities of the measurements, nine subjects were chosen and measured twice (two weeks apart) by two observers. The reliabilities of measurements were assessed with intraclass correlation coefficients (ICC) [10]. The ICC values ranged from 0.03–0.97 in this study.

## Results

Substantial portion of asymptomatic subjects had posterolateral plica of the elbow joints on MRI. The median mediolateral and sagittal dimension of the synovial fold in the asymptomatic group was 3.8 mm and 4.7 mm, respectively (Table 1). Using the dimension criteria of mediolateral dimension, 34 of 50 asymptomatic elbow joints (68%) had the posterolateral plica. In contrast, 42 (84%) elbow joints had the plica in sagittal dimension. Thus, overall, among the 50 asymptomatic elbows, posterolateral plica was observed in 92% (46 elbows) of asymptomatic elbows at least in one of the planes (coronal or sagittal) in MRI images (Table 2). In terms of the coverage of the radial head by the synovial fold, the median mediolateral and sagittal coverage was 16% and 21%, respectively (Table 3). Using the coverage criteria, 18% (nine elbows) had the posterolateral plica at least one plane (coronal or sagittal) on the MRI (Table 4).

**Table 1:** Comparison of mediolateral and sagittal dimensions of the synovial folds between the asymptomatic group and the plica group.

Parameter	Asymptomatic group				Plica group				p-value
	10^th^	50^th^	90^th^	IQ Range	10^th^	50^th^	90^th^	IQ Range	
Mediolateral (mm)	1.9	3.8	5.2	2.8–4.3	5.3	7.0	9.1	6.5–7.9	< 0.001
Sagittal (mm)	1.9	4.7	7.5	3.8–5.9	5.4	7.4	9.2	5.6–8.6	< 0.001

Data are presented using 10^th^, 50^th^, and 90^th^ percent of dimensions of synovial folds. IQ, interquartile range.

**Table 2:** Comparison of proportion of the elbows with posterolateral plica (dimension ≥ 3mm) between the asymptomatic group and the plica group.

	Asymptomatic group	Plica group	p-value
Mediolateral	34 (68)	14 (100)	0.014
Sagittal	42 (84)	14 (100)	0.183
Mediolateral or sagittal	46 (92)	14 (100)	0.568

Data are presented using number and percent in the parentheses.

**Table 3:** Comparison of coverages of radial head by the synovial folds between asymptomatic group and plica group.

Parameter	Asymptomatic group				Plica group				p-value
	10^th^	50^th^	90^th^	IQ Range	10^th^	50^th^	90^th^	IQ Range	
Mediolateral (%)	8	16	24	13–19	22	30	39	24–35	< 0.001
Sagittal (%)	9	21	35	17–28	22	31	41	25–37	< 0.001

Data are presented using 10^th^, 50^th^, 90^th^ percent of coverage amount of radial head by the synovial fold. IQ, interquatile range.

**Table 4:** Comparison of proportion of the elbows with posterolateral plica (radial head coverage by synovial fold ≥ 30%) between the asymptomatic group and the plica group.

	Asymptomatic group	Plica group	p-value
Mediolateral	0 (0)	5 (36)	< 0.001
Sagittal	9 (18)	7 (50)	0.015
Mediolateral or sagittal	9 (18)	9 (64)	0.001

Data are presented using number and percent in the parentheses.

The plica group showed greater dimension of posterolateral synovial fold compared to the asymptomatic group (Table 1). The median mediolateral and sagittal dimension of the synovial fold in the plica group was 7.0 mm and 7.4 mm, respectively. Furthermore, using the dimension criteria, all elbows in the plica group had the posterolateral plica on the MRI in both the coronal and the sagittal planes. However, a substantial portion of elbows (92%) in the asymptomatic group also had the posterolateral plica using the dimension criteria (Table 2). In terms of the coverage criteria, the proportion of the elbows with posterolateral plica in the plica group was significantly greater than that of the asymptomatic group (64% vs. 18%; p < 0.001) (Table 4).

## Discussion

There is no consensus concerning the dimension of the posterolateral synovial fold that can cause symptoms [1, 4, 6, 7, 9]. In the present study, we documented normative data of the dimensions of posterolateral synovial fold in asymptomatic subjects. In addition, we compared the data to those of the patients with posterolateral plica syndrome of the elbow joint. The principal finding of this study was that the dimensions of the posterolateral synovial fold in the plica group were greater than those of the asymptomatic group even though a substantial portion of the asymptomatic subjects also had posterolateral plica.

Our findings do not support the hypothesis that the prevalence of posterolateral plica of the elbow joint is low in asymptomatic subjects. The prevalence of posterolateral plica in asymptomatic subjects was revealed as 86–98% in the previous studies [2, 7]. In a qualitative analysis of the previous study involving 60 asymptomatic subjects, posterolateral plica was evident in 98% of asymptomatic subjects [2]. The authors of the previous study defined the plica when there was an observable meniscoid-like synovial fold [2]. Thus, we initially thought that this prevalence could have been exaggerated because they did not use criterion to define the plica in terms of dimensions. In contrast to our initial thought, the prevalence of the plica of our study was similar with the previous study despite we set the criterion in terms of dimension to define the presence of the plica.

The criteria to diagnose the posterolateral plica have not been established. The previous study suggested that the craniocaudal dimension should be taken into account to diagnose the posterolateral elbow plica because craniocaudal dimension was < 3 mm in most asymptomatic subjects [2]. However, measuring the craniocaudal dimension is less reliable than mediolateral or sagittal dimension. In the previous study, the ICC value of measurements in craniocaudal dimension was only 0.274, which was lower than ICC values in other dimensions (0.871 for mediolateral dimension and 0.702 for sagittal dimension, respectively) [2]. Furthermore, in another study, mean craniocaudal dimension of the plica in cadaveric elbows was only 1.7 mm [7]. Therefore, there has been no consensus about the thickness (craniocaudal dimension) of the plica [6]. We think that, with the craniocaudal dimensions of the synovial fold, it is important that shape (irregular, nodular or blunted tip) and/or inflammatory reaction of the surround tissues should be taken into account for diagnosing posterolateral plica syndrome of the elbow joint [1, 9].

The results of this study support the hypothesis that the plica group has larger dimensions of synovial fold on MRI compared to the asymptomatic group. Most previous studies of the posterolateral plica were clinical case series only including patients underwent arthroscopic plica excision, cadaveric studies, or studies using MRI of asymptomatic subjects. Thus, results of direct comparison of the data between symptomatic and asymptomatic subjects has been lacking in the literature. We directly compared the data between the two groups and realized that the median mediolateral and sagittal dimensions of the synovial fold were larger in the plica group. However, at the same time, MRI criteria suggested by the previous studies did not clearly distinguish the synovial fold which causes symptoms or not. Among the asymptomatic patients, 92% had posterolateral plica when applying the dimension criteria (dimension ≥ 3 mm). Thus, this criterion probably is not appropriate to diagnose symptomatic posterolateral plica syndrome. When using the definition of the coverage of radial head by the synovial fold ≥ 30%, there was difference in the prevalence of the plica between the two groups. However, in one previous study used the same criterion, there was no statistical difference between the patients with and without the posterolateral plica (33% vs. 7%) [9]. Thus, we believe that further study is still needed to study the MR diagnostic criteria of posterolateral plica syndrome of the elbow.

This study has several limitations. First, number of the patients with symptomatic posterolateral plica was small, thus we were not able to calculate the dimension to diagnose posterolateral plica of the elbow joint. However, 14 patients were relatively large compared to other clinical series of posterolateral elbow plica. In addition, combined with the findings of the 50 asymptomatic subjects, we believe that this study can provide valuable information to the readers. Second, all asymptomatic subjects were young males. In contrast, age and sex proportions were different in the plica group. This could have introduced bias in comparison of median dimensions of the synovial fold between the two groups even though the median dimensions of asymptomatic subjects were similar with those of previous study [2]. However, caution is still needed when comparing our findings to other population with different sex compositions.

## Conclusions

The patients who underwent arthroscopic surgery for posterolateral plica syndrome had larger dimensions of the posterolateral synovial fold and higher prevalence of the posterolateral plica on conventional MRI compared to the asymptomatic subjects.

## Supporting information

S1 Data Data of asymptomatic patients. (XLSX)
